# Biostimulation-Based Approaches for Gingival Tissue Augmentation in Thin Periodontal Phenotype: Potential Applications for Orthodontic Patients

**DOI:** 10.3390/jcm15020576

**Published:** 2026-01-11

**Authors:** Amelia Rusiecka, Natalia Bielecka-Kowalska, Sebastian Kłosek

**Affiliations:** 1 Department of Periodontal Diseases and Oral Mucosa Diseases, Medical University of Lodz, Al. Kościuszki 4, 90-419 Łódź, Poland; amelia.rusiecka@outlook.com; 2 Department of Oral Pathology, Medical University of Lodz, Al. Kościuszki 4, 90-419 Łódź, Poland; sebastian.klosek@umed.lodz.pl

**Keywords:** gingival recession, orthodontics, platelet-rich fibrin, low-level light therapy, tissue regeneration

## Abstract

Gingival recession, particularly in patients exhibiting a thin periodontal phenotype, is a prevalent and challenging complication associated with orthodontic treatment, among other factors. Recent advances in biostimulation therapies aim to support soft tissue augmentation by increasing gingival thickness (GT) and keratinized tissue width (KTW) while minimizing the need for invasive surgical procedures. This narrative review explores the available clinical evidence regarding several biostimulation techniques, including injectable platelet-rich fibrin (i-PRF), microneedling (MN), concentrated growth factors (CGF), atelocollagen, hyaluronic acid (HA), and low-level laser therapy (LLLT), with a particular focus on their potential adjunctive role in orthodontic patients with a thin periodontal phenotype. While i-PRF and microneedling—used alone or in combination—have shown promising short-term soft tissue thickening and reduced patient morbidity when compared with conventional grafting procedures, the available evidence is largely derived from small, randomized trials, pilot studies, and non-orthodontic cohorts, limiting the strength of comparative conclusions. Minimally invasive biostimulation techniques may represent potential adjunctive strategies for soft tissue management in selected clinical scenarios. Nevertheless, current evidence remains limited and heterogeneous, and robust, long-term, orthodontic-specific clinical trials are required before these approaches can be considered reliable alternatives to established surgical protocols or validated preventive strategies against gingival recession.

## 1. Introduction

Currently, a significant number of patients are undergoing orthodontic treatment, which not only greatly improves their appearance but also enhances the physiological function of the stomatognathic system. This type of therapy requires careful planning, the ability to anticipate outcomes, and presents numerous challenges for dentists. The capacity to predict potential complications allows for the implementation of preventive measures before they arise. One common complication, particularly in individuals with a thin periodontal phenotype, is gingival recession. Early identification allows preventive measures to be implemented. The periodontal phenotype, encompassing gingival thickness (GT), keratinized tissue width (KTW), and underlying bone morphotype, was historically termed “periodontal biotype” [1,2]. It can be assessed using clinical or radiographic methods, classified as invasive or non-invasive for GT, static or functional for KTW, and bi- or tridimensional for buccal bone plate thickness (BBPT) [2].

A thin periodontal phenotype is characterized by narrow keratinized gingiva, delicate tissue, and limited bone support, increasing the risk of gingival recession, dehiscence, and fenestration. Occlusion type and tooth contact locations also influence periodontal stability by affecting force distribution. GT varies among individuals and regions of the mouth; thin phenotypes are more common in females and Asian populations [3]. Each millimeter of gum recession corresponds to approximately 2.8 mm of bone dehiscence, with contributing factors including periodontal disease, poor oral hygiene, aging, and reduced vascularity and collagen [4].

Orthodontic treatment, whether followed by fixed or removable retention, can exacerbate gingival recession, particularly in thin phenotypes. Mandibular central incisors are especially vulnerable, and labial displacement increases the risk due to limited gingival and alveolar bone thickness [3,5]. About 20–25% of patients may develop recession within 2–5 years post-treatment. In some cases, periodontal surgery may be required, but flap procedures in thin phenotypes can result in shrinkage, often necessitating soft tissue augmentation. Novel biostimulation techniques for gingival augmentation may offer a less invasive solution [2].

This narrative review aims to summarize current evidence on i-PRF, microneedling, CGF, atelocollagen, HA, and LLLT, highlighting their potential in soft tissue management, particularly in the context of orthodontic treatment. Given the limited number of studies in orthodontic cohorts, this work also underscores the need and potential for future research to validate these approaches in this specific patient population.

## 2. Materials and Methods

This article is a narrative, clinically oriented review focusing on biostimulation techniques for gingival augmentation in patients with a thin periodontal phenotype. Emphasis is placed on the potential adjunctive use of these techniques in orthodontic treatment. Rather than following a formal systematic review protocol, this study aimed to integrate current clinical and experimental evidence, highlight recurring patterns and gaps in the literature, and generate hypotheses for future research. An extensive literature search was conducted using PubMed, Scopus and Google Scholar. The search covered publications from 1997 to 2025 and was restricted to English-language articles. Combinations of free-text keywords and abbreviations were used, including ‘injectable platelet-rich fibrin’, ‘i-PRF’, ‘microneedling’, ‘concentrated growth factors’, ‘CGF’, ‘atelocollagen’, ‘hyaluronic acid’, ‘HA’, ‘low-level laser therapy’, ‘LLLT’, ‘gingival augmentation’, ‘gingival thickness’, ‘keratinized tissue’, and ‘thin periodontal phenotype’. Studies were selected based on their clinical or biological relevance to the modification of the gingival or periodontal phenotype, soft tissue biostimulation or adjunctive therapies aimed at increasing gingival thickness (GT) and/or keratinised tissue width (KTW). The review primarily considered randomised controlled trials, cohort studies, pilot clinical trials and in vitro experiments involving human-derived tissues or cells. Studies were selected based on their relevance to the clinical or biological modification of the gingival or periodontal phenotype, soft tissue biostimulation or adjunctive therapies aimed at increasing the thickness of the gingiva (GT) and/or the width of the keratinized tissue (KTW). The review primarily considered randomised controlled trials, cohort studies, pilot clinical trials and in vitro experiments involving human-derived tissues or cells. Information was extracted for each included technique (i-PRF, microneedling, combined i-PRF and microneedling, CGF, collagen-based preparations, hyaluronic acid and laser-assisted approaches) on the study design, population or experimental model, intervention protocol, primary clinical or surrogate outcomes (particularly GT, KTW, periodontal phenotype changes and parameters related to gingival recession), follow-up period and main results. Wherever possible, specific data relating to orthodontic patients or thin periodontal phenotypes was highlighted.

Due to the heterogeneity of study designs, patient populations, intervention protocols, and outcome measures, a formal risk-of-bias assessment and quantitative synthesis were not performed. Instead, the evidence was synthesized narratively, highlighting key trends and findings for each biostimulation modality and organized by intervention type. Attention was to the level of invasiveness compared with conventional grafting procedures, and the feasibility of using these techniques as adjunctive tools in orthodontic soft tissue management. While this narrative approach allows a broad overview of current research, it inherently carries limitations, including potential selection and publication biases, and precludes meta-analysis. Therefore, the results should be interpreted with caution, and further well-designed clinical studies are needed to confirm the observed effects and establish robust clinical guidelines.

The authors used ChatGPT (versions 5, 5.1, and 5.2) during manuscript preparation to improve clarity and assist in generating figures and tables. All outputs were reviewed and approved by the authors, who take full responsibility for the published work.

## 3. Periodontal Phenotype Modification Therapy

In 2016, the Best Evidence Consensus (BEC) introduced a framework aimed at identifying key factors for preserving periodontal and peri-implant health. Their findings emphasize that phenotype modification therapy (PhMT) plays a crucial role in sustaining or enhancing oral health, particularly before undergoing significant restorative or orthodontic procedures. It utilizes various techniques, including free gingival grafts (FGG), CAF, and their combination with subepithelial connective tissue grafts (CTG) or acellular dermal matrices and enamel matrix derivatives, to enhance soft tissue thickness, ultimately improving restorative, orthodontic, and therapeutic outcomes [6,7]. The BEC also examined the characteristics of thick and thin gingival and peri-implant phenotypes, revealing that a thin phenotype is more susceptible to recession and inflammation [6,8]. As summarized in Table 1, PhMT may provide several clinical advantages for patients undergoing orthodontic treatment. Conversely, potential risks and limitations of these interventions are outlined in Table 2, highlighting considerations for clinical decision-making.

Research was carried out to assess the clinical advantages of periodontal PhMT, specifically hard tissue augmentation (PhMT-b) and soft tissue augmentation (PhMT-s), for patients undergoing orthodontic treatment [9].

Particulate bone grafting as part of PhMT-b, combined with corticotomy-assisted orthodontic therapy (CAOT), appears to offer clinical advantages. These include periodontal phenotype modification, preservation or improvement of facial bone thickness, expedited tooth movement, and an increased range of safe tooth displacement in orthodontic treatment. A systematic review concluded that CAOT combined with bone augmentation may offer clinical benefits for orthodontic patients. However, due to the scarcity of research, the effectiveness of PhMT-s alone in orthodontic therapy remains unclear [3,9].

It is worth reviewing the benefits of surgical augmentation methods based on the meta-analysis. According to the studies presented here, there is no clear consensus on the best treatment for GT augmentation or whether the increased thickness is permanent over time, studies have shown that periodontal plastic procedures using autogenous grafts (such as FGG or CTG) or substitutes (such as acellular dermal matrix [ADM] or collagen matrix [CM]), living cellular constructs (LCC) combination with an apically positioned flap (APF) can significantly increase GT [10]. An overview of these procedures, including technique variations, effects on GT and KT, and principal clinical outcomes, is presented in Table 3. Overall, based on this meta-analysis, it can be assumed that these methods using autologous, allogeneic and xenogeneic grafting techniques are effective in increasing KT, GT and overall periodontal health [10].

As the subject of consideration in this article, it should be emphasized that while many grafting techniques effectively increase the volume and quality of soft tissues around teeth and implants, patients often report discomfort at both the donor and recipient sites [7].

## 4. Plateline-Rich Fibrine and Other Autological Blood Product

Autologous blood products, rich in platelets, growth factors, leukocytes, and stem cells, play a crucial role in cell division, collagen synthesis, and angiogenesis, promoting the regeneration and healing of both soft and hard tissues. Incorporating autologous blood products as adjuncts in mucogingival therapy may enhance treatment outcomes, particularly in cases where anatomical or patient-related factors limit the predictability of clinical and esthetic success [7].

The following assumptions can be made about the most common use of blood products—platelet-rich plasma (PRP) is primarily applied in hard and soft tissue treatments, PRF is commonly used for gingival recession as well as furcation and intrabony defect therapies, and CGF is mainly utilized for bone regeneration [11].

PRF is an autologous, bioactive material that contains a fibrin matrix enriched with platelets, leukocytes, and growth factors (e.g., VEGF, PDGF, TGF-β, BMP-1, IGF). Contains a porous fibrin scaffold that facilitates cell adhesion, migration, and differentiation. As the fibrin scaffold gradually breaks down, it slowly releases physiological levels of growth factors. PRF aids in vascular regeneration and promotes the proliferation and migration of osteoblast-related cells, such as mesenchymal cells, osteoblasts, and osteoclasts, while also providing certain immunomodulatory and antibacterial effects—promoting tissue regeneration and healing. Known for its strong osteogenic properties, PRF is widely utilized in bone tissue engineering and dental applications (Table 4 and Table 5) [12].

### Protocol for PRF, PRP, CGF Preparation for Dental Purposes

The preparation PRF for dental use involves several steps to ensure the optimal collection and processing of blood to obtain a high-quality fibrin clot. First, the required materials include blood collection tubes (preferably glass or plastic without anticoagulants), a centrifuge machine with adjustable speed and time, sterile needles and syringes for venipuncture and blood collection, as well as sterile gloves, surgical instruments, and a sterile workspace to maintain aseptic conditions during the procedure. PRP preparation currently lacks standardization and typically involves two centrifugation steps: blood is drawn into tubes with anticoagulants, centrifuged to separate components, then centrifuged again to concentrate platelets, resulting in PPP above and PRP below. PRF is simpler, using one centrifugation without anticoagulants to yield platelet-poor plasma (PPP), PRF, and red blood cells. CGF is also prepared without anticoagulants, using variable centrifugation speeds and times to separate the blood into three layers, with CGF in the middle layer along with some intact platelets [13].

To begin the process, approximately 9–10 mL of venous blood is drawn from the patient using a sterile syringe. It is essential to transfer blood immediately into the collection tubes without anticoagulants to prevent clotting before the centrifugation. The blood should be processed as quickly as possible, ideally within 2 min, to ensure that the fibrin clot forms correctly. Next, the blood is subjected to centrifugation. The centrifuge speed and time depend on the specific type of PRF being prepared (Table 6).

After centrifugation, three distinct layers are formed in the blood collection tube. The top layer is the PPP, which can be discarded or used for mixing with other materials if necessary. The middle layer, known as the PRF clot, is the most important and should be collected for dental use. The bottom layer consists of red blood cells, which should be discarded. The PRF clot can then be removed using sterile tweezers and, if required, compressed to remove excess serum. This can be done by placing the clot on sterile gauze or a PRF box and gently pressing it, particularly if the goal is to create PRF membranes [12].

Fresh PRF must be used immediately as it starts degrading within a few hours. Lyophilized PRF (Ly-PRF) can be stored for longer by freeze-drying, i-PRF should be used within minutes before it polymerizes into a gel (Table 7 and Table 8) [12].

Based on the research, these conclusions can be drawn:

L-PRF, when combined with bone graft materials, showed excellent results in periodontal bone defects, making it the best option for general bone regeneration and implantology.

A-PRF, due to its higher leukocyte concentration, showed better outcomes in soft tissue healing, making it superior for periodontal tissue healing.

i-PRF, in its liquid form, is ideal for injectable use in applications like peri-implantitis and TMJ disorders, making it best suited for non-invasive procedures such as periodontal defects.

T-PRF, providing better mechanical strength and slower degradation, is useful for bone grafting, offering the strongest fibrin network for prolonged effects and ensuring structural stability [12].

## 5. Injectable Platelet-Rich Fibrin

Increasing gingival thickness (GT) with i-PRF has been reported to produce results comparable to those of free gingival grafts (FGGs), the current gold standard, while potentially offering improved aesthetics and reduced postoperative discomfort. These findings highlight the potential of i-PRF as a non-invasive method for gingival augmentation, particularly in patients with a thin gingival biotype. Reviews and studies indicate a significant increase in GT and, in some cases, a wider band of keratinized tissue (KTW) following i-PRF treatment [15]. Comparisons with FGGs suggest that i-PRF may achieve similar outcomes while providing additional patient-centered benefits. Nevertheless, these conclusions should be interpreted cautiously, and further studies with larger sample sizes and longer follow-up periods are needed to establish standard clinical guidelines [15].

A clinical study assessing the effect of i-PRF on gingival phenotype reported a statistically significant increase in GT at both individual sites and tooth level, with GT increasing by 26.6% at 3 months and 29% at 6 months compared to baseline. No significant changes were observed in KTW, suggesting that i-PRF may primarily enhance tissue thickness [16].

A meta-analysis evaluating changes in GT and KTW after i-PRF injection in patients with a thin gingival phenotype found a significant increase in GT. Regarding KTW, results varied depending on the number and interval of injection sessions: a significant increase was observed after four sessions at 10-day intervals, whereas three sessions at 7-day intervals resulted in a non-significant increase [17].

Finally, a split-mouth randomized controlled trial on a small patient group compared multiple i-PRF injections with hyaluronic acid. Both treatments led to increases in GT and KTW, with no statistically significant differences between the methods. Both minimally invasive approaches appeared more effective in increasing GT than KTW. The authors suggested that repeating this study in a larger population could help confirm these findings and strengthen the clinical evidence [18].

Further studies suggest that the addition of i-PRF may enhance the effectiveness of surgical treatment. In the reported trial, sites treated with CAF + CTG + i-PRF (with CTG immersed in i-PRF liquid for 15 min) demonstrated greater improvements in recession depth reduction and keratinized tissue height compared to those treated with CAF+CTG alone, indicating a potential beneficial role of i-PRF in improving clinical outcomes (Table 9) [19].

## 6. Microneedling and Its Role in Gingival Biostimulation

As mentioned earlier, subepithelial connective tissue grafts among PhMTs are widely considered the gold standard for gingival augmentation. However, these procedures require advanced surgical skills and are often associated with poor patient compliance. Meanwhile, percutaneous collagen induction therapy, now commonly known as micro-needling (MN), has emerged as an innovative and minimally invasive option for dermatological, but perhaps also periodontal, treatment [6].

MN also referred to as percutaneous collagen induction therapy, is a modern dermatological technique that facilitates skin renewal by creating microscopic injuries. These controlled microchannels initiate the wound healing process—comprising inflammation, proliferation, and remodelling—while causing minimal harm to the epidermis. This stimulation encourages the production of essential growth factors, including platelet-derived growth factor, fibroblast growth factor (FGF), and transforming growth factors (TGFa and TGF-b). As a result, fibroblast activity enhances neovascularization and collagen production, gradually transitioning Type III collagen into Type I over a period of weeks to months, leading to firmer skin and a reduction in wrinkles. Additionally, MN has been reported to enhance the expression of type I collagen genes, glycosaminoglycans, and several growth factors, including vascular endothelial growth factor, FGF-7, and epidermal growth factor. Histological studies conducted up to a year after treatment have suggested increases in collagen within the reticular dermis, greater elastic fiber content, epidermal thickening, and maintenance of overall skin structure. Some research also indicates that MN may promote TGF-β3 production, which could contribute to tissue regeneration and potentially reduce fibrotic scarring by influencing the balance between TGF-β3 and the fibrosis-associated TGF-β1 and TGF-β2. [20].

It is worth noting that MN can also be used in combination with surgical techniques, yielding satisfactory outcomes. When combined with CAF, MN has been suggested as a potential graft-free approach for increasing GT, with some studies reporting outcomes that may be comparable to those achieved with CAF plus acellular dermal matrix; however, further well-designed clinical studies are needed to substantiate these observations. Additionally, this combination may offer a potentially greater improvement in KTW in the treatment of RT1 recession defects in patients with a thin gingival phenotype [21].

## 7. Injectable Platelet-Rich Fibrin and Microneedling

MN involves creating tiny wounds that result in slight superficial bleeding, initiating a natural healing process that releases a variety of growth factors. The effect of MN on neoangiogenesis helped thicken the gingiva, while i-PRF promoted an increased release of growth factors. Studies indicate that after 10 days, i-PRF releases growth factors such as epidermal growth factor (EGF), insulin-like growth factor-1 (IGF-1), and various forms of platelet-derived growth factor (PDGF) at a higher rate than PRP [22].

The application of i-PRF, both independently and in combination with microneedling, has been suggested as a reliable and safe method for addressing the thin gingival phenotype. A notable increase in GT was observed, with MN alongside i-PRF demonstrating possibly improved results. Incorporating this approach prior to periodontal plastic surgery or orthodontic treatment could potentially enhance the success of these interventions [1]. The combination of MN and i-PRF offers a minimally invasive, non-surgical approach to enhancing GT. Notably, incorporating i-PRF as an additional element led to better results in increasing tissue thickness compared to using MN alone [6]. According to Yadav et al., MN may offer certain advantages over i-PRF in modifying the phenotype of individuals with a thin periodontal profile, given the reported greater increase in gingival thickness (GT) and the benefit of avoiding autologous blood collection [23]. Valli et al. compared the clinical effects of i-PRF and MN with free gingival grafting (FGG) for periodontal phenotype modification, finding that both approaches resulted in significant increases in GT and keratinized tissue width (KTW) at 6 months, with no significant differences between groups. The combination of i-PRF and MN was associated with less discomfort and higher patient-reported aesthetic satisfaction. These findings suggest that non-invasive treatment with i-PRF + MN could achieve clinical outcomes comparable to more invasive protocols while potentially improving patient well-being. However, these conclusions should be interpreted with caution, as they are based on a small prospective clinical trial, and a more critical appraisal of both esthetic and functional outcomes is warranted until further studies confirm these findings. [24].

From a practical perspective, the combined use of i-PRF and microneedling appears potentially feasible in routine clinical settings, as both techniques are minimally invasive and can be performed chairside with commonly available equipment. However, their adoption in everyday practice may be influenced by factors such as the need for blood collection and centrifugation for i-PRF preparation, operator training in microneedling techniques, chair time, and patient acceptance. In addition, the lack of clear standardization and clinical guidelines currently limits widespread clinical implementation. Therefore, while the approach shows promise, its routine use in general dental practice should be considered cautiously until clearer clinical guidelines become available.

## 8. Concentrated Growth Factors (CGF) in Periodontal Regeneration

CGF is a third-generation platelet-rich derivative prepared using a unique acceleration and deceleration centrifugation method. It contains a denser collagen matrix, higher tensile strength, and increased cytokine concentration, making it an advanced material for bone and soft tissue regeneration. The CGF is prepared from autologous blood. A sample of the SHO is separated into PPP, CGF-rich gel, and red blood cells. The CGF layer includes fibrin-rich, erythrocyte-containing, and leukocyte-rich zones. This process enhances platelet rupture, boosts growth factor release, and forms a dense fibrin matrix resistant to degradation. In both injectable and membrane forms, CGF enhances soft tissue healing. Injectable CGF promotes fibroblast proliferation and reduces oxidative stress, while the gel form provides a physical scaffold at surgical sites. CGF contains a rich mix of growth factors, including PDGF, TGF-β1, VEGF, IGF-1, EGF, b-FGF, and BMPs. These factors collectively stimulate fibroblast proliferation, angiogenesis, epithelial regeneration, and extracellular matrix formation—key processes in gingival tissue repair [25]. Studies suggest CGF may improve outcomes when used alone or combined with biomaterials such as collagen matrices or bone substitutes, enhancing graft integration and prolonging growth factor activity [25]. The current evidence does not definitively position CGF as superior to PRF; it can be considered either an alternative or a complement, depending on clinical objectives and practitioner preference. Further comparative clinical studies are needed to clarify their specific role (Table 10 and Table 11).

CGF is commonly applied in oral surgery, primarily to support hard tissue regeneration. It may also enhance the viability and overall quality of grafts [11]. Though current clinical evidence is still limited, CGF is a promising biostimulant for gingival augmentation as a logical extension based on CGF’s proven effects on fibroblasts, vascularization, and soft tissue regeneration in the face and oral region. Its autologous origin, regenerative properties, and potential for sustained biological activity position it as a valuable adjunct in periodontal and mucogingival therapy [25].

Unlike CGF, which has a highly variable centrifugation method, PRF preparation is simpler and its quality more controllable. Additionally, PRF contains physiological levels of growth factors and platelets, which are more conducive to tissue regeneration, while CGF’s supraphysiological concentrations may cause tissue edema and negative cellular responses. As an emerging tissue regeneration material, it can effectively stimulate the regeneration of local bone and soft tissues [12]. Its use in therapy requires further observation and research, particularly large-scale in vivo studies; therefore, the conclusions currently available should be interpreted with caution. Moreover, the scientific evidence regarding the use of CGF remains limited, and additional basic and clinical studies are needed to better understand its biological characteristics and clinical applications.

## 9. Collagen Preparations

Collagen used in biomaterials can be sourced from humans or animals, or produced via recombinant methods. Animal-derived collagen is extracted through pepsin digestion or acid solubilization, resulting in atelocollagen or tropocollagen. Atelocollagen is commercially favored due to reduced cross-species antigenicity associated with telopeptides. Consequently, both allogenic and xenogenic collagens are generally considered safe for use [26]. The mechanical behavior of connective tissues is closely linked to their specific functions, and any alterations can result in dysfunction or disease. In most mammalian tissues, this mechanical environment is shaped primarily by the micro- and nanoscale structure of collagen and its interactions with surrounding components. The recently described tropocollagen spring mechanism reveals that collagen fibrils in certain tissues can undergo significant elongation under minimal load [27]. Tropocollagen, the fundamental building block of collagen fibers, plays a crucial role in maintaining the structural integrity of periodontal tissues.

The use of tropocollagen for gingival augmentation, particularly as a preventive approach to recession during orthodontic treatment in individuals with a thin periodontal phenotype, remains poorly documented. However, in vitro studies indicate that collagen-based biomaterials composed of tropocollagen can promote tissue regeneration by enhancing cell adhesion, proliferation, and extracellular matrix formation [28]. Collagen scaffolds loaded with human periodontal ligament fibroblasts have shown potential in increasing soft tissue volume and strength, suggesting a role for tropocollagen-derived materials in stabilizing gingival tissues in orthodontic patients [28]. Further research is needed to clarify its direct clinical applications.

Injectable equine atelocollagen, initially used in aesthetic medicine, has demonstrated promise in gingival augmentation by stimulating fibroblast activity and supporting soft tissue regeneration. Clinical reports indicate that as few as two injections can improve gingival condition, reduce recession height, enhance papillae, and increase tissue thickness. Collagen also contributes to blood clotting and regulates mucosal physiology through interactions with hyaluronic acid and enamel matrix proteins [26]. Atelocollagen has further been shown to reduce bleeding on probing (BOP), as reported in patients with Hashimoto’s disease. Injections administered into keratinized gingiva at two-week intervals produced the most significant reduction after the first two applications, with slower improvement thereafter. These findings support the potential role of atelocollagen in biostimulatory therapies for gingival augmentation and periodontal health [29]. Beyond periodontology, collagen-based biomaterials demonstrate regenerative potential across various medical fields. For instance, a combination of atelocollagen and octacalcium phosphate granules has been successfully applied to alveolar clefts in patients with unilateral cleft lip, promoting bone bridge formation and eruption of adjacent permanent teeth within six months postoperatively [30]. In otolaryngology, experimental scaffolds combining PRP with atelocollagen and silicone sheets have shown high success rates in closing chronic tympanic membrane perforations, although further validation is needed [31].

In vitro studies suggest that medical compounds containing type I collagen can promote an anabolic phenotype in tenocytes, stimulating proliferation, migration, and collagen synthesis, potentially supporting tendon repair. Rigorous clinical studies are necessary to determine the true potential of these compounds [32]. Volume-stable collagen scaffolds also promote angiogenesis and maintain high cell turnover, comparable to native periodontal ligament tissue, and have proven effective in supporting periodontal regeneration, particularly in intrabony defects [33]. Atelocollagen sponges combined with recombinant human basic fibroblast growth factor have enabled rapid healing of full-thickness skin wounds unresponsive to growth factors alone, highlighting their regenerative potential in gingival augmentation [34].

Collagen-based membranes and scaffolds have been integrated into surgical approaches for localized gingival recession. Methods combining root preparation, surgical flap advancement, and collagen membranes—with or without fibrin sealing systems—have demonstrated effective tissue regeneration [35]. Xenogeneic collagen matrices, composed of dense and spongy layers providing space for clot formation, have been shown to treat gingival recession effectively; however, connective tissue grafts still provide superior outcomes in increasing GT and promoting phenotypic conversion [36]. Recent studies on injectable porcine type I collagen devices indicate promising results in supporting gingival regeneration. In vitro experiments demonstrated increased fibroblast viability, accelerated wound closure, and activation of key mechanotransduction markers (FAK, YAP, TAZ), suggesting that injectable collagen can serve as an effective mechanical scaffold for tissue repair [37]. Nonetheless, these findings are largely supported by in vitro or small-scale studies; therefore, they should be interpreted with caution until further in vivo and clinical trials are conducted. Proper injection technique is essential to minimize risks, such as vascular compromise, and small, low-pressure injections with aspiration are recommended [38].

## 10. Laser-Assisted Gingival Augmentation

Laser therapy has attracted increasing scientific interest; however, the current body of evidence remains limited. As a result, firm or definitive conclusions cannot yet be drawn regarding its clinical effectiveness, safety, or the durability of the observed outcomes. Potential complications and long-term effects are still not fully understood. Nevertheless, selected scientific reports are presented below to provide insight into the current state of knowledge and to illustrate possible directions for future research in this field. Most studies have investigated the use of laser therapy as an adjunctive intervention, while research on laser therapy as a stand-alone biostimulatory method remains limited, which may reflect the current constraints of this approach.

Systematic reviews evaluated and compared studies on the use of lasers as monotherapy or as an adjunct to surgical periodontal treatment. Evidence suggested that lasers may improve clinical outcomes and reduce morbidity in non-surgical periodontal therapy, owing to their ablation, vaporization, hemostasis, and sterilization effects. However, in flap surgery with or without laser use, no statistically significant difference in the primary outcome was observed (Lasers considered in the review: carbon dioxide (CO2), neodymium-doped yttrium, aluminum and garnet (Nd-YAG), erbium, yttrium, aluminum and garnet (ErYAG) and diode lasers). Laser use in flap surgery or guided tissue regeneration (GTR)/enamel matrix derivative (EMD) showed no significant improvement in outcomes. GTR/EMD alone performed better than laser treatment, but differences were minor. Overall, evidence does not support lasers as effective adjuncts, and results should be interpreted cautiously due to limited data and high variability [39].

The CAF technique is commonly favored for treating gingival recession, especially when adequate attached gingiva is present, and incorporating a laser-assisted vestibular releasing incision may further enhance surgical outcomes. Platelet use as an adjunct has shown positive results, but post-surgical laser biostimulation remains unstudied. In a study comparing CAF with PRF and laser-assisted CAF with PRF (additional vestibular release incision using diode laser and post-photobiomodulation using diode laser—980 nm and 1 Watt power in continuous mode/60 s), both groups showed significant improvements at six months, except in probing depth and root coverage. In this study, also no significant differences were found between groups, highlighting the need for further research on the benefits of laser-assisted CAF [40]. In another research, effectiveness of flap surgery combined with laser therapy was evaluated against flap surgery alone for treating gingival recession. Meta-analysis showed that the addition of laser therapy offered clinical benefits in keratinized tissue width and in probing depth (PD) and clinical attachment level (CAL) at a 1-year follow-up. However, the combination did not improve root coverage or aesthetic outcomes. Further long-term studies are needed to evaluate these aspects [41].

Effectiveness of low-level laser therapy (LLLT) in enhancing clinical and patient-centered outcomes of CTG procedures for gingival recession is still uncertain. Four randomized clinical trials were analyzed: two reported significantly better results with LLLT, while the other two found no difference between LLLT and CTG alone. High variability was observed between groups regarding gingival recession depth and KTW. Meta-analysis revealed a statistically significant improvement in gingival recession depth with LLLT combined with CTG compared to CTG alone. Nonetheless, the limited number of studies and variation in laser parameters call for cautious interpretation of these findings [42]. Moreover, study indicates that low-level laser therapy may enhance the percentage of complete root coverage when used in combination with CTG. This was evaluated in a study comparing the treatment of gingival recession using CTG alone versus CTG combined with low-level laser therapy (CTG + L) [43].

Recent research highlights the growing use of lasers to enhance wound healing, though further in vitro studies are needed due to ongoing debate over their role in soft tissue regeneration. One study compared the effects of various doses of Er:YAG and Er,Cr:YSGG lasers on human gingival fibroblast (HGF) proliferation. Both lasers significantly increased HGF proliferation at 24 and 48 h compared to controls, with the highest increase observed after 30 s of irradiation. These findings suggest that Er:YAG and Er,Cr:YSGG lasers may promote soft tissue healing by stimulating cell proliferation [44]. However, this statement is not supported by clinical evidence.

The study examined the effectiveness of adjunctive photobiomodulation in enhancing outcomes after applying the laterally closed tunnel technique to treat isolated gingival recession. Primary and secondary outcomes included changes in recession depth, recession width, KTW, periodontal biotype, pain levels (VAS), and early wound healing (EHS index). The results indicated that while photobiomodulation did not significantly improve short-term (3-month) recession coverage, it did enhance early healing and patient comfort. Further research is needed to evaluate its long-term effects on recession coverage [45].

## 11. Hialuronic Acid and Membranes

The study assessed the effectiveness of using HA as an adjunct in treating gingival recession with the CAF technique. Findings suggest that its local application does not offer additional clinical benefits [46]. In contrast, another study evaluated the use of amnion membrane and 0.2% HA gel as adjuncts in root coverage procedures. Results indicated that, when combined with the CAF technique, both were safe and led to statistically significant improvements, with the experimental group showing better clinical outcomes than the control group [47]. Other novel approaches involve placing a chorion membrane during periodontal pocket treatment, which may help increase GT in regions with a thin gingival biotype [48].

Overall, while some evidence suggests that HA may provide added benefits when used alongside surgical treatment for labial gingival recession, the findings across studies are inconsistent. Its effect on CAL gain in intrabony defects remains unclear, and reported outcomes vary depending on the specific adjunct and protocol used [49]. Although HA appears safe with no reported adverse effects, the high variability among studies and limited sample sizes highlight the need for further well-designed randomized controlled trials to clarify its true clinical potential. Moreover, although both HA and injectable platelet-rich fibrin (i-PRF) have been investigated as adjuncts in periodontal and gingival augmentation procedures, direct comparisons between the two remain scarce, and further research is needed to evaluate their relative efficacy (Table 12).

## 12. Conclusions

Innovative biostimulation techniques, such as i-PRF and microneedling, show potential as adjunctive approaches for gingival augmentation in orthodontic patients with a thin periodontal phenotype. Current evidence suggests these minimally invasive procedures may provide short-term increases in gingival thickness. Additionally, they offer perspectives for soft tissue augmentation, recession coverage, and broader aspects of tissue regeneration due to shared underlying mechanisms.

The use of blood-derived products has several practical advantages, being relatively inexpensive and straightforward to apply. However, their use may be limited in patients with certain ethical or moral considerations, although this also provides an alternative for individuals who do not accept animal-derived materials. Commercially available products may incur additional costs, which should be considered. Operator skill is another important limitation, highlighting the need for qualified personnel to perform these procedures under strict sterile and safety conditions.

While many methods show promising potential, the current evidence remains largely hypothesis-generating and requires further clinical confirmation. Future studies should also address aspects such as cost-effectiveness, learning curves, and regulatory or ethical considerations to better inform clinical decision-making.

Further research is required to explore the potential use of tropocollagen, atelocollagen, CGF, HA, and LLLT in biostimulation and gingival augmentation. These techniques are still considered experimental but may provide valuable adjuncts to existing methods, offering new perspectives for enhancing GT and preventing recession in patients with a thin periodontal phenotype.

Available data are predominantly short-term, heterogeneous, and frequently derived from non-orthodontic cohorts or in vitro studies. This limits the ability to draw firm conclusions regarding long-term outcomes, prevention of recession, or periodontal phenotype modification. Consequently, clinical recommendations must remain cautious. Well-designed, long-term, orthodontic-specific studies are essential to validate efficacy, clarify mechanisms of action, and assess practical applicability.

This review highlights the potential of biostimulation therapies and underscores the need for further research to provide robust evidence for their use in orthodontic soft tissue management. It goes beyond a descriptive summary by critically considering the quality, consistency, and clinical applicability of current data.

Several biostimulation modalities, including i-PRF, microneedling, CGF, HA, and LLLT, demonstrate promising outcomes, particularly for increases in GT and, to a lesser extent, KTW. However, findings across studies are heterogeneous and sometimes conflicting. For instance, although LLLT and HA have been associated with improved soft tissue healing or marginal clinical gains in certain investigations, other randomized trials failed to demonstrate additional benefit compared with conventional surgical techniques alone. Similarly, CGF shows strong biological plausibility and encouraging preliminary outcomes, but available data are predominantly early-phase or indirect.

Methodological differences between studies further contribute to inconsistency, including variation in study design, patient populations, follow-up duration, and assessment methods for soft tissue outcomes. GT and KTW were not measured uniformly across investigations, with differences in techniques, reference points, and clinical thresholds, limiting inter-study comparability. This underscores the need for future trials to adopt consistent and validated measurement protocols when assessing periodontal phenotype modification.

Although this review emphasizes orthodontic patients with a thin periodontal phenotype, much of the current evidence comes from non-orthodontic or mixed populations. Therefore, the orthodontic relevance of many reported outcomes remains largely inferential. The biological rationale supporting potential benefit in orthodontic settings is strong, particularly regarding enhancement of soft tissue resilience and potential protection against recession. Nonetheless, well-designed, orthodontic-specific clinical trials with adequate long-term follow-up are essential before these biostimulation modalities can be considered reliable adjuncts or preventative strategies in orthodontic care.

## Figures and Tables

**Table 1:** Possible Advantages of PhMT in Orthodontic Treatment [3].

Possible Advantages of PhMT in Orthodontic Treatment	
Improved periodontal health	through dentoalveolar augmentation, leading to increasing GT and KTW, which helps prevent future gingival recession and attachment loss during orthodontic movement.
Greater stability of orthodontic results.	
Lower risk of periodontal complications	especially gingival recession and attachment loss, in certain orthodontic cases.
Faster orthodontic treatment duration.	
Enhanced periodontal and orthodontic results	due to improved tissue and bone support.
Wider range of treatment options	for managing dentofacial misalignments.
Potential decrease in the need for extractions	in Class II malocclusion cases with crowding that would typically require orthognathic surgery.
Reduced reliance on orthodontic camouflage	reduced compromises during decompensation
Possible increase in oral cavity volume	by optimizing bone volume, which can allow for broader limits of arch expansion.

**Table 2:** Potential Risks and Limitations of PhMT in Orthodontic Treatment [3].

Potential Risks of PhMT in Orthodontic Treatment	Limitations of PhMT in Orthodontic Treatment
Root damage during surgical procedures.	Acceptance challenges both the dental community and patients, due to potential additional adverse effects and the cost of periodontal procedures.
Pulpal devitalization as a potential complication.	Increased complexity in interdisciplinary case management, requiring more oversight for a successful outcome.
Minor papillary recession may occur in some cases.	Higher cost and longer treatment duration, with the possibility of multiple surgical procedures, especially in cases with very thin, soft tissue, where soft tissue augmentation is needed before corticotomy and bone augmentation. This adds to both cost and surgical complexity.
Infection risks associated with dentoalveolar surgeries.	Need for orthognathic surgery in some cases with skeletal discrepancies, even after PhMT, to achieve the optimal results.

**Table 3:** Gingival phenotype modification therapies [10].

Procedure Type	Techniques Analyzed	Gingival Thickness (GT)	Keratinized Tissue (KT)	Key Findings
Root Coverage Procedures	ADM, CM, CTG	Significantly increased GT compared to flap alone	Only CTG and ADM significantly increased KT	All techniques improved GT. CTG and ADM significantly improved KT. Early GT predicted future recession.
Non-Root Coverage Procedures	ADM, CM, FGG, LCC with APF	Not analyzed	All treatments (ADM, CM, FGG, LCC) increased KT compared to APF alone	KT increased overtime, with sustained GT augmentation.
Conclusion for Root Coverage	CTG, ADM	All graft materials significantly enhanced GT	CTG and ADM significantly enhanced KT	CTG and ADM are superior for both GT and KT in root coverage procedures.

**Table 4:** Effects of PRF on Various Biological Processes [12].

Biological Process	Effects of PRF
Angiogenesis	PRF promotes neovascularization by upregulating key angiogenic factors (e.g., VEGF, PDGF) and activating intracellular signaling pathways.
Osteogenesis-Related Cells	PRF supports osteogenesis through activation of multiple osteogenic signaling pathways.
Osteoblasts	Enhances osteoblast proliferation and differentiation, upregulates OPG expression, and promotes mineralization.
Osteoclasts	Inhibits osteoclastogenesis, suppresses RANKL-induced differentiation, and promotes osteoclast apoptosis.
Immunomodulatory	Reduces inflammation by modulating macrophage polarization (promoting M2 over M1) and regulating immune responses.
Antimicrobial Mechanisms	Exhibits antibacterial activity through release of hydrogen peroxide and antimicrobial peptides can also serve as a drug delivery scaffold.

**Table 5:** Applications of PRF in Dentistry [12].

Application	Effect
Alveolar Ridge Preservation	PRF reduces alveolar bone resorption and promotes bone healing post-extraction.
Guided Bone Regeneration	Enhances biocompatibility of bone graft materials and improves their osteogenic potential.
Maxillary Sinus Floor Elevation	PRF is used alone or with bone substitutes to enhance bone formation in sinus augmentation procedures.
Periodontal Infrabony Defect Repair	Stimulates periodontal ligament regeneration and improves clinical attachment levels.

**Table 6:** Protocol of PRF and CGF preparation [13,14].

PRF TYPE	Centrifuge Speed and Time	Special Conditions
SUPERFICIAL PLATELET-POOR PLASMA (PPP)	Top layer above PRF or CGF clot	Can be discarded or used for mixing with other materials if necessary.
PLATELET-RICH PLASMA (PRP)	5600 rpm; the upper layer is transferred and centrifuged again at 2500–3000 rpm	With anticoagulant.
ADVANCED PRF (A-PRF)	100× *g* for 14 min; 1500 rpm, 14 min	Contains more leukocytes and promotes better healing.
INJECTABLE PRF (I-PRF)	60× *g* for 3 min	Stays in liquid form, suitable for mixing with biomaterials.
HORIZONTAL PRF (H-PRF)	700× *g* for 8 min	Uses horizontal centrifugation for better cellular distribution.
TITANIUM PRF (T-PRF)	2700 rpm for 12 min	Uses titanium tubes for better fibrin quality.
STANDARD PLATELET-RICH FIBRIN (S-PRF)	2700 rpm for 12 min	Dense fibrin clot with minimal interfibrous space.
CONCENTRATED GROWTH FACTORS (CGF) CONCENTRATED LIQUID	accelerate for 30 s, then 2700 rpm for 2 min, 2400 rpm for 4 min, 2700 rpm for 4 min, 3000 rpm for 3 min, finally, decelerate for 36 s and stop—middle layer	Without anticoagulant. Acceleration and deceleration repeated centrifugation.

**Table 7:** Characteristics of Different Generations of PRF and pEVs [12].

Generation	PRF Type	Centrifuge Process	Composition	Special Characteristics	Advantages	Limitations
First-Generation– Contains platelets but lacks a fibrin network	Platelet-Rich Plasma (PRP)	Requires anticoagulants, centrifugation at 160–250× *g* for 10–15 min	Platelets, plasma proteins, few leukocytes	Lacks fibrin matrix; mainly provides platelet-derived growth factors	Enhances healing, promotes angiogenesis	Requires exogenous activation, short-term release of growth factors
Second-Generation– Forms a fibrin matrix with trapped growth factors and cells, prolonging their release	Leukocyte- and Platelet-Rich Fibrin (L-PRF)	No anticoagulants, centrifuged at 400× *g* for 12 min	Fibrin network with leukocytes, platelets, growth factors (VEGF, PDGF, TGF-β)	Dense fibrin scaffold, sustained release of bioactive molecules	Improves wound healing, promotes bone regeneration, better handling properties	Requires immediate use, difficult to store
	Advanced PRF (A-PRF)	Lower speed (100× *g* for 14 min)	Similar to L-PRF but with increased leukocyte content	Enhanced release of growth factors over time	Greater angiogenic potential, improved cell proliferation	May have batch variability
	Injectable PRF (i-PRF)	Low-speed centrifugation (60× *g* for 3 min)	Liquid form, no fibrin clot	Can be injected directly into defects or mixed with biomaterials	Easy application, enhances cell migration and vascularization	Short-lived, must be used immediately
	Horizontal PRF (H-PRF)	Horizontal centrifugation (700× *g* for 8 min)	Similar to L-PRF but with smoother separation of blood components	Uniform distribution of growth factors and cells	More consistent fibrin structure, improved handling	Limited clinical studies
	Titanium PRF (T-PRF)	2700 rpm for 12 min using titanium tubes	Similar to L-PRF but with denser fibrin network	Higher biocompa- tibility, increased growth factor release	Enhanced mechanical stability, better tissue integration	Requires specialized centrifuge tubes
Third-Generation	Concentrated Growth Factor (CGF)	Multi-step centrifugation (acceleration/deceleration phases, 2400–3000 rpm)	Higher fibrin density, increased cytokine levels	More robust fibrin structure, improved mechanical strength	Greater stability, higher growth factor concentration	Complex preparation process, variability in composition
Platelet Derived Extracellular Vesicles (pEVs)	pEVs	Ultracentrifugation (300× *g* to 100,000× *g*)	Nanoparticles with growth factors, nucleic acids (mRNA, miRNA), and mitochondria	Highly bioactive, involved in intercellular communication	Potential for targeted therapy, high regenerative potential	Difficult to isolate, requires advanced processing techniques

**Table 8:** Comparison of PRF Types for Dental Applications [12].

PRF Type	Best Dental Applications	Advantages	Limitations
L-PRF (Leukocyte-PRF)	Periodontal regeneration, bone grafting, implantology, sinus lifts, guided bone regeneration (GBR)	High fibrin density, prolonged release of growth factors, promotes bone regeneration and angiogenesis.	Requires immediate use, rapid degradation, lower injectability.
A-PRF (Advanced PRF)	Periodontal defects, soft tissue healing, alveolar ridge preservation	Increased leukocyte content, higher release of growth factors, enhances soft tissue healing.	Longer centrifugation time, may have variability in composition.
i-PRF (Injectable PRF)	Periodontal regeneration, peri-implantitis treatment, TMJ therapy, regenerative endodontics	Remains liquid for easy injection, enhances cell migration and vascularization.	Short-lived, must be used within minutes.
T-PRF (Titanium PRF)	Bone regeneration, alveolar ridge preservation, sinus augmentation	Denser fibrin network, improved mechanical stability, enhanced drug-loading capacity.	Requires specialized titanium tubes for preparation.

**Table 9:** Summary and comparison of i-PRF studies [15,16,17,18,19].

Study (Author, Year)	Study Design	Intervention	Population	Key Findings on GT	Key Findings on KTW	Other Outcomes
Manasa et al., 2023 [16]	Split-mouth RCT	i-PRF	General population	GT increased by 26.56% (3 months) and 29% (6 months)	No significant changes in KTW	i-PRF effective for gingival phenotype modification
Idris et al., 2024 [17]	Systematic review and meta-analysis	i-PRF injections (3 vs. 4 sessions)	Thin gingival phenotype	Significant GT increase in all cases	Significant KTW increase with 4 sessions (10-day intervals); non-significant with 3 sessions (7-day intervals)	Dose/frequency influenced outcomes
Faour et al., 2022 [18]	Split-mouth RCT	i-PRF vs. Hyaluronic Acid	Thin gingival phenotype	Both increased GT, no significant difference	Both increased KTW, but less than GT	Both techniques were minimally invasive and effective
Żurek et al., 2024 [15]	Systematic review of RCTs	i-PRF vs. FGG	General population, thin gingival biotype	i-PRF significantly increased GT; results comparable to FGG	In some cases, wider KTW observed	i-PRF offered better aesthetics and less postoperative discomfort
Ucak Turer et al., 2020 [19]	RCT	CAF + CTG vs. CAF + CTG + i-PRF	Patients with deep gingival recessions	Both groups improved; greater RD reduction in i-PRF group	Greater KTW increase in i-PRF group	88% root coverage with i-PRF vs. 80% without; difference not statistically significant

**Table 10:** Growth factors contained in CGF [25].

Growth Factor	Function	Relevance to Gingival Regeneration
PDGF (Platelet-Derived Growth Factor)	Stimulates fibroblast and mesenchymal stem cells (MSC) proliferation; promotes angiogenesis and collagen synthesis	Enhances fibroblast migration and extracellular matrix (ECM) production in gingiva
TGF-β1 (Transforming Growth Factor β1)	Stimulates MSCs, epithelial cells, and Schwann cells; synergizes with platelet-derived growth factor	Promotes epithelialization and ECM biosynthesis
VEGF (Vascular Endothelial Growth Factor)	Stimulates endothelial cell proliferation and vascular permeability	Promotes angiogenesis for graft vascularization
IGF-1 (Insulin-like Growth Factor 1)	Promotes cell proliferation, chondrogenesis, and neurogenesis	Supports fibroblast viability and collagen regeneration
EGF (Epidermal Growth Factor)	Stimulates epithelial and fibroblast proliferation	Critical for soft tissue healing and epithelial closure
b-FGF (Basic Fibroblast Growth Factor)	Angiogenic and mitogenic for osteoblasts	Supports new vessel formation in gingival tissue
BMPs (Bone Morphogenetic Proteins)	Induce bone and cartilage formation	May support adjacent alveolar bone regeneration

**Table 11:** Application and form of CGF usage [25].

Application Type	CGF Form Used	Mechanism/Outcome
Injectable CGF	Liquid phase	Direct stimulation of fibroblasts and epithelial cells; wrinkle reduction, tissue plumping
CGF Membrane/Gel	Solid phase	Serves as a scaffold in graft sites; protects wound; supports epithelial closure
Combination with Biomaterials	CGF + Collagen/Chitosan/Bio-Oss	Extended release and synergistic healing; potentially ideal for gingival and periodontal regeneration

**Table 12:** Summary table [1,2,3,4,5,6,7,8,9,10,11,12,13,14,15,16,17,18,19,20,21,22,23,24,25,26,27,28,29,30,31,32,33,34,35,36,37,38,39,40,41,42,43,44,45,46,47].

Technique	Effectiveness for GT/KTW	Invasiveness	Cost	Availability	Level of Evidence
CTG/FGG/ADM/CM (conventional grafting, PhMT-s)	High GT and KT gain; CTG/ADM superior for combined GT/KT; effects generally stable long-term	High—flap elevation, graft harvesting, donor-site morbidity	High (surgical time, graft/matrices, operating setting)	Moderate–High in specialist periodontal practice	High—multiple RCTs and meta-analyses in non-orthodontic cohorts
i-PRF (injectable PRF)	Consistent GT increase; KTW improvement protocol-dependent (more sessions → greater KTW)	Low–Moderate—venipuncture and multiple mucosal injections, no flap	Moderate—centrifuge, disposables, chair time	Moderate—requires PRF equipment and trained staff	Moderate—several RCTs and one meta-analysis, mostly short-term and non-orthodontic
Microneedling (MN)	GT increase (sometimes KTW), especially in thin phenotype; results comparable to some grafting adjuncts in selected indications	Low—minimally invasive microperforations, no flap, no donor site	Low—inexpensive devices, limited consumables	High—widely available in dermatology and increasingly in dentistry	Low–Moderate—few periodontal RCTs, follow-up generally short
i-PRF + MN (combined biostimulation)	GT and KTW increase; non-inferior to FGG in short-term, with improved comfort and aesthetics	Low–Moderate—combination of injections and MN; no surgical grafting	Moderate—PRF setup plus MN, but no graft material	Moderate—requires both PRF system and MN devices	Low–Moderate—limited number of RCTs/prospective trials, short-term outcomes only
CGF (concentrated growth factors)	Promising for soft-tissue regeneration (angiogenesis, fibroblast proliferation); direct evidence for GT/KTW still limited	Low–Moderate—venipuncture; often used as adjunct in surgical sites	Moderate—specific centrifugation protocol, equipment	Moderate—available in some surgical/implant centers	Low—mainly preclinical and early clinical data; very limited gingival-specific trials
Injectable atelocollagen/tropocollagen-based preparations	GT increase and bleeding reduction reported in pilot studies; effects on KTW not well defined	Low—local injections, no flap or graft harvesting	Moderate–High—branded medical devices, repeated sessions	Limited–Moderate—available in aesthetic medicine; sparse periodontal use	Low—pilot clinical studies, case reports, extrapolation from other indications
Collagen membranes/xenogeneic collagen matrix (surgical)	KT increase and some GT gain; generally inferior to CTG for phenotypic conversion but avoids donor site	Moderate–High—flap surgery, membrane placement	Moderate–High—membrane cost plus surgical time	High in periodontal/implant practice	Moderate—several RCTs and controlled trials in recession coverage and intrabony defects
Hyaluronic acid (HA; injections/gels, often with CAF)	Modest GT/KTW and CAL benefits; some studies show added effect vs. surgery alone, others no additional gain	Low–Moderate—topical application or injections, usually adjunct to surgery	Moderate—commercial products, repeated applications	High—widely available dental/medical product	Low–Moderate—small RCTs and one systematic review, heterogeneous protocols
LLLT/photobiomodulation (often adjunct to CTG/CAF or tunnels)	May improve early healing and recession depth/CAL in some studies; effect on final root coverage and KTW inconsistent	Very Low—non-contact light application, no additional tissue trauma	High (equipment); Low per session—device-dependent	Moderate—present in some periodontal and surgical offices	Low–Moderate—few RCTs, high variability in parameters, meta-analyses with cautious conclusions
Laser-assisted flap/GTR adjuncts (non-LLLT)	Small additional benefits for PD/CAL/KTW in some studies; no clear superiority for root coverage	High—combined with conventional flap/GTR surgery	High—surgical plus laser equipment	Moderate—limited to laser-equipped centers	Low–Moderate—heterogeneous studies, limited high-quality data in recession coverage

## Data Availability

No new data were created or analyzed in this study.
